# Implant Survival of an Uncemented Modular Femoral Implant in Patients With Severe Femoral Bone Loss and 2-Stage Hip Revision

**DOI:** 10.1155/2024/6158822

**Published:** 2024-10-25

**Authors:** Sebastian Winther, Naima Elsayed, Karen Dyreborg, Elinborg Mortensen, Michael. M. Petersen, Jens Stürup, Nikolaj S. Winther

**Affiliations:** ^1^Department of Orthopedic Surgery, Rigshospitalet, Copenhagen, Denmark; ^2^Surgical Centre, Landssjúkrahúsið, Tórshavn, Faroe Islands; ^3^Department of Clinical Medicine, Faculty of Health and Medical Sciences, University of Copenhagen, Copenhagen, Denmark

## Abstract

**Background and Purpose:** Revision total hip arthroplasty (rTHA) is a challenging procedure especially in the presence of severe bone loss where implant fixation is compromised. The aim of this study was to evaluate implant survival, clinical outcome, and midterm results in a group of complex patients after femoral revision using an uncemented modular implant design.

**Patients and Methods:** We performed a retrospective study including 100 patients (101 hips) treated with revision THA using an uncemented modular implant design. We identified 51 hips as Paprosky types I-II and 50 hips as Paprosky III-IV bone defects. We reviewed operative reports and radiographs. Patients underwent a clinical examination to assess the Harris Hip Score (HHS) and completed patient-reported outcome measures (PROM), including the Oxford Hip Score (OHS) and the EQ-5D Visual Analog Scale (VAS). Minimum follow-up was 2 years (average, 5.8 years; range, 2.0–9.4 years).

**Results:** Among the cases, 46 hips were revised for infection in a 2-stage procedure and 44 hips for aseptic loosening. 11 hips had periprosthetic fractures of Vancouver type B2 or B3. A total of 5 hips required revision with removal of the femoral implant and 11 patients experienced complications resulting in fracture (*n* = 1), dislocation (*n* = 10), and soft tissue revision (*n* = 1). The 5-year implant survival estimated by Kaplan–Meier survival analysis was 95% (95%-CI: 91%–99%). All hips had radiographic evidence of osseointegration and no one with subsidence greater than 5 mm. Additionally, 80% showed radiographic evidence of restoration of proximal femoral bone. Mean HHS was 78.

**Conclusion:** In complex cases of revision THA, using a modular revision femoral system yielded promising results. The 5-year implant survival estimated by Kaplan–Meier survival analysis was 95% (95%-CI: 91%–99%), with all hips demonstrating radiographic evidence of osseointegration and no one with subsidence greater than 5 mm. Notably 80% showed radiographic evidence of restoration of proximal femoral bone.

## 1. Introduction

Revision total hip arthroplasty (rTHA) is a challenging procedure especially in the presence of severe bone loss that compromises implant fixation. The incidence of primary total hip arthroplasty (THA) has been increasing over the last decades and overall long-term results are excellent [1]. However, due to the large volume of patients, younger patients undergoing the THA procedure, prolonged life expectancies, and extended indication areas [1, 2], there is an increasing requirement for revision surgery [3, 4].

One of the prominent surgical challenges is achieving stable and durable fixation for revision femoral implants, especially when dealing with substantial femoral bone loss, poor bone stock, and altered femur geometry. Especially periprosthetic joint infection (PJI) and multiple procedure-related factors in 2-stage exchange arthroplasty are associated with severe bone loss [5, 6].

When dealing with poor proximal femoral bone, the surgeon is reliant on achieving distal fixation, the goal being a stable durable fixation with preservation and restoration of bone stock.

Tapered fluted stem and long cylindrical stems offer some advantages and have showed promising options for uncemented femoral revision [7, 8] also in situations with periprosthetic fracture [9–11]. The advantage of the diaphyseal or distal fixation implants lies in their ability to bypass areas of proximal bone deficiency and provide stability and fixation in the more distal femoral bone [8, 12, 13]. Both mono-block and modular femoral implants have been used for the past twenty years to address femoral revision cases [14]. However, uncemented modular implants provide surgeons the flexibility to adjust leg length and offset and apply the ideal version of the neck to address stability [2, 12].

To date, few studies with a small number of patients have been published regarding outcomes after modular femoral implants in patients undergoing hip revision surgery for large bone defects and after 2-stage revision [9, 15, 16], and there remains a need for more comprehensive investigations. Therefore, the aim of this study was to assess the survival of the implant, clinical outcome, and midterm results after femoral revision with an uncemented modular implant design in a complex group of patients.

## 2. Materials and Methods

We performed a retrospective study reviewing 100 consecutive patients (101 hips) who underwent a rTHA. The procedures were performed using the Arcos Modular Revision Femoral System (Zimmer Biomet, Warsaw, IN, USA). Patients were included between September 2011 and December 2018, all treated at a tertiary referral medical center for hip revision surgery at Rigshospitalet (Copenhagen, Denmark) on any indication. Bone loss was categorized preoperatively as Paprosky types I–IV [17] and cases with periprosthetic fracture were also classified according to the Vancouver system [18].

Baseline demographics are presented in Table 1.

**Figure 1:**
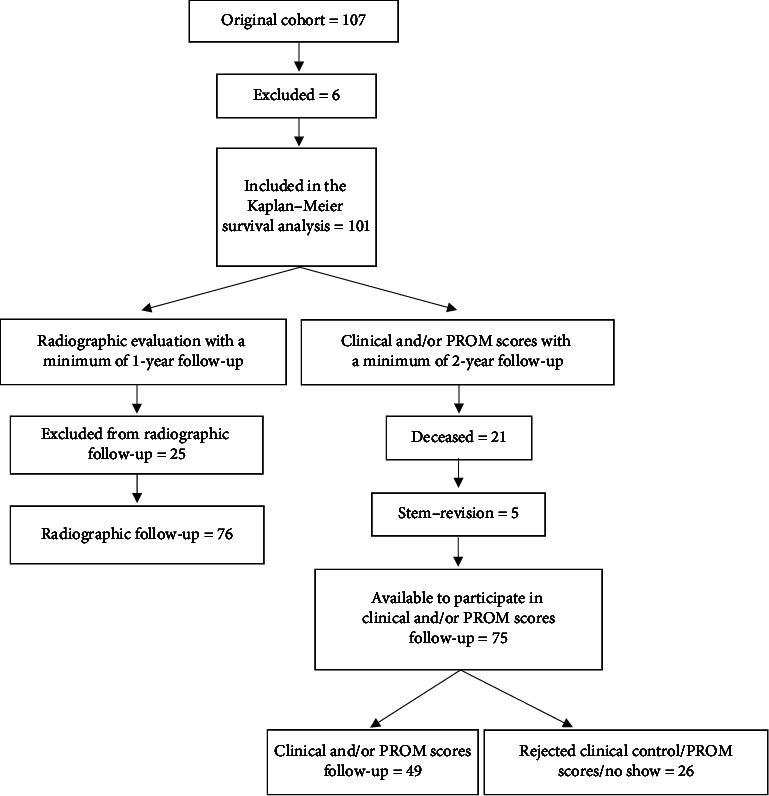
Flowchart of patients included in the study.

Patients were identified through the operation database (Sundhedsplatformen) by searching for all operations accomplished by three senior surgeons who performed revision THA. We identified 106 patients (107 hips). Of these, 6 patients were excluded. Four of those were excluded because revision procedures of both the hip and knee joint were performed (total femur prothesis). One patient was excluded due to being revised 7 days after index surgery caused by the Arcos stem penetrating the anterior cortex, and the patient was revised with another nonporous coated screw fixated stem. One patient was excluded because of a cardiac arrest and hip dislocation with removal of the implant 2 days after index surgery and no new femoral implant was implanted. Consequently, the Kaplan–Meier survival analysis was based on 101 hips from 100 patients (Figure 1).

All patients were routinely evaluated within 12 weeks after surgery and at 1 year. Further, patients in this study were invited to a recent follow-up, where all patients received AP radiographs of the pelvis and true-lateral radiographs of the hip, if no recent X-rays already existed. Clinical assessment included evaluation by using HHS [19] and PROM using OHS [20] and EQ-5D [21]. The EQ-5D consists of two parts and only the VAS score is reported in this article. The minimum follow-up for clinical and patient reported outcome measures was 2 years (mean: 5.8 years; range, 2.0–9.4 years). Of the 100 patients, 49 patients were available for clinical assessment and/or PROM scores. Patients living on the Faroe Islands or Greenland (*n* = 8) filled in the PROM scores and had radiographic follow-up, but no HHS was obtained due to logistic challenges.

Radiographic assessment was performed on digitized images by one consultant orthopedic surgeon (NW). The preoperative, immediate postoperative, and most recent radiographs were reviewed evaluating bone loss, osseointegration of the distal femoral stem, subsidence, and restoration of the proximal femur. Implants were considered radiographically unstable in the presence of a circumferential lucency around the implant or with subsidence greater than 5 mm [22]. Osseointegration was assessed as per the criteria of Engh et al. [23] that describe “fixation by bone ingrowth” as an implant without subsidence and minimal or no radiopaque line formation around the stem. Subsidence was assessed by measuring the difference between the most proximal point of the greater trochanter and the proximal point of the modular taper on standing AP radiographs. If this difference exceeded 5 mm, it was noted, indicating instability of the implant [24]. In hips where the greater trochanter could not be used as a fixed bony point, the lesser trochanter or another fixed point was used as an alternative landmark [24, 25]. The measurements were calibrated according to the known head size of the femoral prosthesis.

Femoral restoration was classified subjectively as per Kolstad et al. as no bone regeneration, possible regeneration, and definite new bone formation [26].

We excluded 25 out of 101 hips in the radiographic follow-up due to patients with less than 1-year radiological follow-up or poor-quality radiographs with inconsistencies in femoral rotation, precluding a proper comparison from fixed anatomical landmarks or patients. The minimum follow-up for radiographic follow-up was 1 year. The mean radiographic follow-up was 3.5 years (range 1–9.4 years).

All study participants provided informed written consent about personal and medical data collection prior to study enrollment. The Danish Patient Safety Authority granted access to patient files for those patients whom we were unable to contact (case number (3–3013-1695/1), and the local Data Protection Agency of the Capital Region of Denmark approved the study (RH-2017–336, I-suite nr.: 05999).

### 2.1. Implant

Arcos revision system is a modular press-fit titanium revision stem design that supplies the surgeon with numerous combinations of proximal bodies and distal stem to reconstruct defects commonly seen in femoral revision surgery.

There are three types of stems applied for all revisions in this study: a fluted straight tapered stem (STS) and a long cylindrical curved stem with or without distal screw fixation. All three stems strive for instant load and distal fixation. The STS aims for immediate distal fixation around the conical stem transfer load distally and provides rotational stability. The long cylindrical curved stems aim for fixation around the isthmus bone and depend on the amount/scale of bone loss.

The proximal body comes in different lengths, diameters, and two offset options (standard and high offset). This provides the possibility of adjustment after stem placement and reproduces various patient anatomies and leg lengths, thus optimizing prosthesis contact area. The choice of implants was planned preoperatively by templating and finally decided by the surgeon intraoperatively. A trochanteric reattachment plate allows screw fixation of the trochanteric fragment directly to the implant increasing stability. This option was used in cases with extended trochanteric osteotomy (ETO) and in cases with an unstable trochanteric bone often seen in periprosthetic fracture Vancouver type B3.

### 2.2. Statistics

The statistical analysis software used in this study was SPSS (IBM SPSS Statistics, version 25) and RStudio (Version 1.2.1335 2009-2019 RStudio, Inc.).

Unless otherwise specified, all reported values are presented as mean and range. Survival analysis was performed with the end point defined as revision of the femoral component for the Arcos hip for any reason. The Kaplan–Meier survival analysis was used to calculate the possibility of implant survival of the femoral component. All patients were followed until their most recent follow-up, revision, or death. Patients who died or were lost to follow-up were included in the implant survivorship analysis. The 95% confidence intervals (CIs) for the cumulative 5 year survival were calculated. Kaplan–Meier survival analysis was also used to calculate the possibility of implant survival of the patients.

## 3. Results

All hips (*n* = 46) undergoing revision due to infection had a two-stage procedure, with the definitive implant being implanted at the second stage. Forty-five (*n* = 45) of the revisions involved replacement of a cemented prothesis and 46 replacements of an uncemented prothesis. Bone loss was categorized preoperatively and we found 51 hips with Paprosky I-II bone loss and 50 hips as Paprosky types III-IV bone defects. 11 patients with periprosthetic fracture all had loose implants and Vancouver type B2 and type B3 fracture (Table 2).

**Figure 2:**
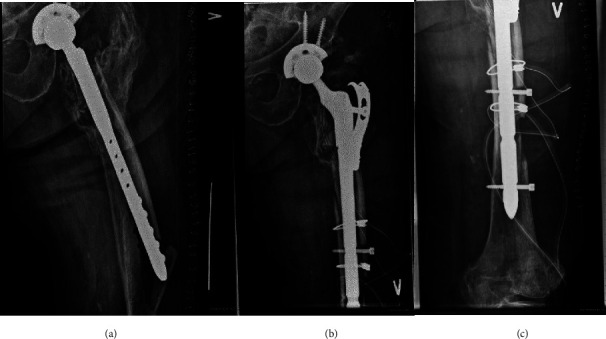
Radiographs showing (a) preoperative anterior to posterior view of the left femur in Vancouver B3 periprosthetic fracture of an uncemented femoral component; (b–c) rTHA using an Arcos cylindrical modular femoral implant with a trochanter reattachment plate and distal screw fixation.

Out of the total 101 hips, 30 had long cylindrical implants. There was a tendency toward selecting long cylindrical implants in the most severe cases where the isthmus area was compromised using the option to fixate the implant below the isthmus with locking screws (illustrated in Figure 2).

In 6 of the 7 cases who had nonsupportive diaphysis bone defect (Paprosky type 4 defect), a distal screw fixated stem was used. In these cases, tapered fluted stems were not sufficient in obtaining distal fixation.

In 73 hips, a total revision involving both the acetabular and femoral implants was performed, while 28 hips underwent a femoral stem revision only. In those patients where the acetabulum implant was revised, an uncemented highly porous coated acetabular component was implanted and screws were fixated to secure initial mechanical stability. The articulation was in all cases 32 mm metalheads in an elevated highly crosslinked polyethylene liner. No dual mobility devices or constraint liners were used.

**Figure 3:**
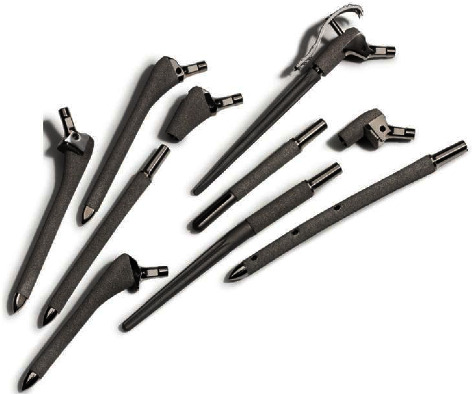
The Arcos modular femoral revision system (with permission from Zimmer Biomet).

**Figure 4:**
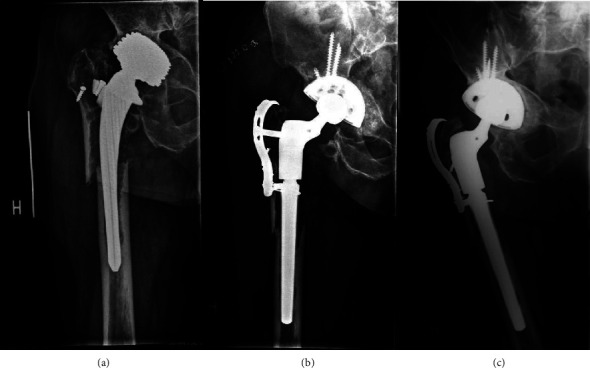
Radiographs showing (a) preoperative anterior to posterior view of the right femur with aseptic loosening and fracture of an uncemented femoral component and Paprosky type IIIB bone loss; (b) rTHA using an Arcos tapered, modular femoral component with a with a trochanter reattachment plate; (c) 2 years follow-up showing a well fixed osseointegrated Arcos component with restoration of proximal femoral bone stock.

Eleven hips were revised because of a periprosthetic fracture with a loose implant and were classified as Vancouver type B2 (*n* = 3) and type B3 (*n* = 8) fractures. In cases of periprosthetic fracture type B3 and in cases of severe bone loss resulting in an unstable greater trochanter, a reattachment plate was used for reattachment of the trochanteric bone directly to the implant (Figures 2, 3, and 4). In cases where ETO was performed, titanium cerclage was used to stabilize the osteotomy.

### 3.1. Implant Survival, Rerevisions, and Complications

The probability of the 5-year implant survival estimated by Kaplan–Meier survival analysis based on 101 hips from 100 patients was 95% (95%-CI: 91%–99%)

**Figure 5:**
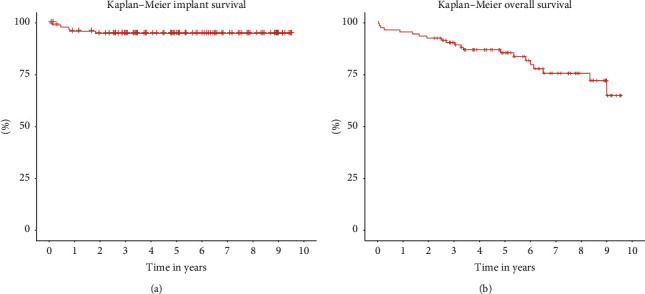
(a) Kaplan–Meier survival analysis probability of implant survival. (b) Kaplan–Meier survival analysis probability of overall survival.

The 5-year overall survival of patients estimated by Kaplan–Meier survival analysis based on 100 patients was 85% (95%-CI: 49.6%–84.2%) (Figures 5(a) and 5(b)).

In total, 5 hips required rerevision with removal of the femoral implant. Of those, 3 patients had their implant removed because of infection. All 3 infected hips had a history of earlier periprosthetic infection and were revised in a 2-stage procedure. 2 of these patients ended with a permanent Girdlestone status. At the time of rerevision, all the infected hips were well integrated in the femoral canal and required an ETO for implant removal. The survivorship for 2-stage revision in this study was 94% and failures were all associated with reinfection.

The periprosthetic fracture group had 100% survivorship for implant revisions and there were no complications recorded.

One patient had the implant removed because of aseptic loosening with subsidence > 5 mm and lack of osseointegration due to a large residual cement mantle left in the femoral canal preventing osseointegration. One patient had an early dislocation turning the stem in retroversion and was revised with a new long cylindric implant with distal screw fixation. 1 patient suffered from a periprostatic fracture around the knee treated with open reduction internal fixation (ORIF) surgery, became infected, and ended with a total femur prosthesis.

Of the 101 hips, 11 patients had complications resulting in fracture (*n* = 1), dislocation (*n* = 10), and soft tissue revision (*n* = 1) with a mean time of 458 days (range 9–1980 days) after surgery.

### 3.2. Radiographic Follow-Up

Of the 101 hips in this study group, there were comparable pre- and postoperative and follow-up radiographs for 76 hips. Of the 76 hips, all had radiographic evidence of osseointegration and no one with subsidence greater than 5 mm. Of the 76 hips, 61 hips (80%) showed radiographic evidence of restoration of proximal femoral bone (Figures 4(a), 4(b), and 4(c)). In cases where an ETO was performed during revision surgery; all showed evidence of healing.

### 3.3. Clinical Follow-Up and PROM Scores

The clinical evaluations showed mean HHS at 78 points (range 41–100). There were 31 patients with excellent or good HHS score (63%), 9 patients with fair score (18%), and 9 patients with poor scores (19%). In our group with 2-stage revision, the HHS score was 78.

The mean OHS results (48 being the best) was 35 points (range 8–48) divided into 35 patients with satisfying or mild to moderate decreased hip function (71%), 9 patients with moderate to severe decreased hip function (18%), and 5 patients with severe decreased hip function (10%). The mean EQ-5D VAS score was 70 (range 25–100).

## 4. Discussion

The increasing demand for hip revision surgery in the presence of severe proximal femoral bone loss and periprosthetic fracture represents a complex reconstructive challenge. Also, infection is associated with severe bone loss as a result of multiple procedures, debridement, and 2-stage procedures [5, 6]. The modular distal fixated stems have demonstrated to improve outcome in these cases by fixation in more distal femoral bone, enrich osseointegration and restoration of proximal bone, and have shown greater than 95% survivorship in second-stage revision for infection [27–30].

Our series included a complex group of patients in which 46% was revised in 2-stage revision due to infection and 50% had severe proximal femoral bone loss (Paprosky III-IV) and were therefore dependent on distal fixation. The overall survivorship was 95% with a mean follow-up of 5.8 years. This is comparable with other studies describing the use of a modular femoral stem design [9, 28–30].

Despite being a difficult reconstructive challenge, our study group shows promising clinical outcomes. The mean HHS was 78 points. This result is comparable with similar studies; Abdel et al. [29] reported a mean HHS of 76, and in a study by Marfo et al. [30], the HHS was 72. Houdek et al. [27] reviewing 57 patients undergoing femoral revision in the setting of two-stage reimplantation reported a mean HHS of 76. In our group with 2-stage revision, the HHS score was 78.

Three patients had their stem removed because of infection and had all history of earlier infection. At the time of rerevision, all 3 hips were well integrated in the femoral canal and required an ETO for stem removal. This highlights the potential of osseointegration as reported in several studies [15, 31, 32]. Amanatullah et al. [15] reported similar result in a series of 92 patients with severe femoral bone loss and found 91 of 92 stems (99%) with radiographic evidence of osseointegration. Amanatullah et al. also had 3 stems removed for infection, all well-fixed.

Patients in our series in whom the stem had not been revised all had radiographic evidence of osseointegration.

Subsidence of the femoral stem is a frequently reported complication in hip revision surgery and may lead to instability, aseptic loosening, and compromised hip function. The reason for early subsidence is probably due to the lack of early press-fit sufficient to withstand patient loading [33–35]. To avoid subsidence, a solid fixation of the implant into the femoral isthmus of a minimum of 2–4 cm is recommended [33, 36, 37].

It has also been reported that cylindrical stems are less stable and less resistant to rotational forces and display more subsidence in the setting of large bone loss and in cases with severely compromised diaphyseal bone, cylindrical stems may not always be a reliable solution. Studies have shown that mechanical failure increased to 21% when this component was used in patients with Type IIIB or Type IV defects [14, 38]. In fact, the one patient in our study that was revised 2 weeks after index revision had a cylindric nonscrew fixated stem. During dislocation, the stem rotated into retroversion and was revised with a new cylindric stem with distal screw fixation.

Therefore, adequate reaming is required for adequate scratch fit cortical contact and is crucial to obtain axial and rotational stable fixation. In our series, the threshold for using nonscrew fixated cylindric stem was more than 4 cm of isthmus bone remaining.

The stem geometry (tapered/cylindric) and length were chosen by the surgeon prior to surgery depending on the region of bone loss. There was a tendency toward selecting long cylindric stem in the most severe cases where the isthmus area was severely compromised using the option to fixate the stem below the isthmus with locking screws. Out of the total of 101 hips, 30 had a long cylindric stem. We found no cases of implant subsidence greater than 5 mm and the use of cylindric stem did not compromise the distal fixation in our study. In contrast, we found it very useful to have the possibility to implant a cylindric stem with distal locking screw in cases of most severe bone loss and useful in dealing with periprosthetic fracture (Vancouver B2 and B3) (illustrated in Figure 2).

In 6 of the 7 cases who had nonsupportive diaphysis bone defect (Paprosky type 4 defect), the distal screw fixated implant was used. In these cases, tapered fluted stems were not sufficient in obtaining distal fixation. No subsidence was seen in any of these cases.

Several studies have shown excellent result using Arcos STS in Vancouver B2 and B3 periprosthetic fracture and greater than 95% survivorship with infrequent subsidence [10, 39–41]. In our study group, 10% (*n* = 11) suffered a periprosthetic fracture with loose stem and Vancouver type B2 (*n* = 3) and type B3 (*n* = 8) fracture. At follow-up, all had a retained implant and there was no complication or subsidence recorded in this group. Patients in the B3 group had a supplementary trochanteric reattachment plate that allows for reattachment of the trochanteric fragment directly to the implant increasing stability and securing abductor muscle attachment on the trochanteric bone improving hip function and stability. This feature is, to our knowledge, unique for the Arcos revision system.

We recorded a dislocation rate of 10% (*n* = 10), a result we considered to be acceptable and relatively low compared with other studies [9, 28–30]. We believe that the trochanteric reattachment plate securing abductor muscle attachment is part of the reason for this result.

Amanatullah et al. [15] reported that instability was the most common complication in their series, accounting for 19% of all cases. They argued that the high dislocation rate was related to the inclusion of patients with severe proximal femoral bone loss and compromised attachments of the abductor musculature. This is an issue when dealing with recurrent revision surgery and proximal bone loss.

Weiss et al. [12] also found a dislocation rate at 19% in a series of 90 femoral revisions. In 8% of the patients, this was limited to only 1 dislocation.

## 5. Conclusion

In this group of patients undergoing complex femoral revisions with the use of a modular revision femoral system, we observed an impressive 5-year implant survival rate of 95% (95% CI: 91%–99%), as determined by Kaplan–Meier survival analysis. Notably, all hips displayed radiographic evidence of osseointegration, with no instances of subsidence greater than 5 mm. Furthermore, 80% showed radiographic evidence of proximal femoral bone restoration. In cases where tapered fluted implants were insufficient for achieving distal fixation, we found it very useful to have the option of using a cylindric implant with distal locking screw. The results highlight the versatility of the modular femoral revisions systems, which can be used effectively in a wide range of complex femoral revision cases, including those involving significant bone loss and periprosthetic fractures.

## Figures and Tables

**Table 1:** Baseline demographics.

	All (*n* = 101)
Alive/dead	80
Sex	
Male/female	50/50
Mean age at surgery in years (range)	69.5 (24–91)
BMI (range)	27.7 (19–41.3)
ASA	
1	8
2	52
3	36
4	3
Previous hip revisions before index	
No revision	46
1 revision	39
2 or more	16
Indication for index revision	
Aseptic loosening	44
Septic loosening	46
Periprosthetic fracture	11

**Table 2:** Paprosky classification.

	All (*n* = 101)	*N* (%)	Stem type∗
Paprosky classification			
Type I	12	11.9	9/2/1
Type II	39	38.6	31/8/0
Type III	32	31.7	24/7/1
Type IV	18	17.8	7/4/7

^∗^STS/cylindrical/cylindrical with interlocking screws.

## Data Availability

The data presented in this study are restricted due to patient confidentiality and are, therefore, not publicly available. However, they are available from the corresponding author on reasonable request.
